# Bilateral upper limb robot-assisted rehabilitation improves upper limb motor function in stroke patients: a study based on quantitative EEG

**DOI:** 10.1186/s40001-023-01565-x

**Published:** 2023-12-19

**Authors:** Congzhi Tang, Ting Zhou, Yun Zhang, Runping Yuan, Xianghu Zhao, Ruian Yin, Pengfei Song, Bo Liu, Ruyan Song, Wenli Chen, Hongxing Wang

**Affiliations:** Department of Rehabilitation Medicine, Zhongda Hospital Southeast University, Nanjing, 210009 China

**Keywords:** Stroke, Bilateral upper limb robot-assisted rehabilitation, Motor function, Activities of daily living, Quantitative electroencephalography

## Abstract

**Background:**

Upper limb dysfunction after stroke seriously affects quality of life. Bilateral training has proven helpful in recovery of upper limb motor function in these patients. However, studies evaluating the effectiveness of bilateral upper limb robot-assisted training on improving motor function and quality of life in stroke patients are lacking. Quantitative electroencephalography (EEG) is non-invasive, simple, and monitors cerebral cortical activity, which can be used to evaluate the effectiveness of interventions. In this study, EEG was used to evaluate the effect of end-drive bilateral upper extremity robot-assisted training on upper extremity functional recovery in stroke patients.

**Methods:**

24 stroke patients with hemiplegia were randomly divided into a conventional training (CT, *n* = 12) group or a bilateral upper limb robot-assisted training (BRT, *n* = 12) group. All patients received 60 min of routine rehabilitation treatment including rolling, transferring, sitting, standing, walking, etc., per day, 6 days a week, for three consecutive weeks. The BRT group added 30 min of bilateral upper limb robot-assisted training per day, while the CT group added 30 min of upper limb training (routine occupational therapy) per day, 6 days a week, for 3  weeks*.* The primary outcome index to evaluate upper limb motor function was the Fugl-Meyer functional score upper limb component (FMA-UE), with the secondary outcome of activities of daily living (ADL), assessed by the modified Barthel index (MBI) score. Quantitative EEG was used to evaluate functional brain connectivity as well as alpha and beta power current source densities of the brain.

**Results:**

Significant (*p* < 0.05) within-group differences were found in FMA-UE and MBI scores for both groups after treatment. A between-group comparison indicated the MBI score of the BRT group was significantly different from that of the CT group, whereas the FMA-UE score was not significantly different from that of the CT group after treatment. The differences of FMA-UE and MBI scores before and after treatment in the BRT group were significantly different as compared to the CT group. In addition, beta rhythm power spectrum energy was higher in the BRT group than in the CT group after treatment. Functional connectivity in the BRT group, under alpha and beta rhythms, was significantly increased in both the bilateral frontal and limbic lobes as compared to the CT group.

**Conclusions:**

BRT outperformed CT in improving ADL in stroke patients within three months, and BRT facilitates the recovery of upper limb function by enhancing functional connectivity of the bilateral cerebral hemispheres.

## Introduction

Upper limb dysfunction is a common problem after stroke, mainly manifested by muscle weakness, spasticity, poor coordination and loss of use on the hemiplegic side. Surveys show that 85% of patients have upper limb dysfunction from the onset and only 1/3 of hemiplegic patients recover partial function of the upper limb [1, 2]. Restoration of upper limb function is one of the main goals of stroke rehabilitation.

Due to its high intensity, repetitive, and task-oriented nature, upper limb robot-assisted therapy (RT) can provide repetitive stimulation training and specific task training of certain intensities for stroke patients, thus promoting the recovery of upper limb function by increasing multiple sensory inputs [3]. Current rehabilitation robots are differentiated according to their contact with the human body and end type, with the exoskeleton type being more common. The end type robot can provide resistance or assistance through the joystick letting the patient achieve active, assisted, or passive movement, and it can be set up and adjusted for patients of different body types, making it more clinically applicable.

Several studies show that RT significantly increases Fugl-Meyer Assessment (FMA) scores and sensory function compared with regular rehabilitation after treatment [3, 4]. These potential advantages of RT are considered useful for sensorimotor recovery in poststroke patients. Unilateral robot-assisted therapy (URT) may be an alternative to conventional therapy, however, some studies have shown that URT is not superior to conventional therapy for activities of daily living in stroke patients within six months [5–7]. In studies using fMRI, a bilateral robot-assisted forearm training system was found to increase interhemispheric connectivity (sensorimotor area) and intrahemispheric connectivity (ipsilateral supplementary motor area to M1 area) [8], promoting functional recovery of the upper limb.

Bilateral upper limb robot-assisted therapy (BRT) supports training in any combination of axes and planes, providing multiple movement patterns (i.e., active, passive, and assisted activities) with interactive training. Studies have confirmed that 10% of the nerve fibers in the cerebral cortex and spinal cord do not cross and control ipsilateral movements, therefore activities of the non-affected upper limb can affect movements of the affected upper limb [9]. At the same time, many daily functional activities depend on the coordinated activity of the bilateral upper limbs, and studies have suggested there is an interaction between the bilateral upper limbs in sensory and motor integration, and that bilateral training with the involvement of the non-affected upper limb is important for functional activity recovery in stroke patients [10]. Studies have pointed out that activation of cortical areas of the brain is higher in patients with bilateral limb movements than with movements of the affected limb alone [11]. Previous studies have noted that this synergistic modality improves upper limb function in patients and may be associated with increased ipsilateral cortical excitability [12–15].

Quantitative electroencephalography (QEEG) has the advantages of simple, non-invasive, and dynamic monitoring. It is sensitive to abnormalities in brain metabolism and neurological function, making it one of the most important means of detecting brain function [16]. Resting EEG is widely used for functional assessment after stroke, with several bands being commonly used including *δ*, *θ*, *α*, *β* and *γ*. *α* activity at rest reflects the level of consciousness and visual attention [17], and lower *α* band energy is the strongest independent predictor of poor functional prognosis [18]. *β* activity has been shown to be associated with pyramidal and inhibitory neurons, which are associated with muscle activity [19–21]. EEG electrode-based functional connectivity (FC) correlates directly with neural activity in the brain, and can be used to predict motor recovery [22]. Until now, the neural correlation has been unclear.

To better understand brain reorganization of sensory-motor areas induced by BRT after stroke, we used BRT to study the effects of upper limb functional recovery and brain reorganization in stroke patients. We hypothesized that BRT improves sensory and motor control after stroke compared to routine occupational therapy. In addition, we applied quantitative EEG to analyze changes in intra- and inter-regional responses after BRT.

## Methods

### Study design and subjects

This study was conducted at the Affiliated Zhongda Hospital of Southeast University from 01 April 2021 to 28 February 2022. 24 patients were randomly assigned to two groups (Conventional group and Bilateral group) in a 1:1 ratio using a simple randomization procedure. The protocol was approved by the Committee of Institutional Ethics (No:2021ZDSYLL091-P01) and registered in the China Clinical Trial Registration Center (ChiCTR2100049484). All patients signed the informed consent.

The inclusion criteria were as follows: (i) diagnosis of stroke was confirmed by computed tomography (CT) and/or magnetic resonance imaging (MRI); (ii) aged 18–80 years old, clear consciousness, able to complete the corresponding assessment (Mini-Mental State Examination (MMSE) ≥ 20 points); (iii) first onset, course of 2 weeks to 3 months; (iv) the modified Ashworth spasticity assessment of the upper limb major muscle tension was less than or equal to grade 2; (v) Brunnstrom assessment of hemiplegic upper limb was in stage II–IV; (vi) stable clinical condition and good control of chronic diseases; (vii) the treatment methods of this study were understood and cooperated. The criteria for exclusion were (i) previous history of brain injury, brain tumor or other neurological diseases; (ii) pain or dysfunction of both upper limbs caused by other reasons; (iii) patients with severe diseases including the cardiovascular system, cerebrovascular system, digestive system, endocrine system, and psychosis; (iv) lesions in cerebellum or brainstem.

### Bilateral upper limb rehabilitation robot equipment

The bilateral upper limb robot-assisted system consists of two upper limb robot-assisted devices (Burt, ESTUN Inc., Nanjing, China) connected by a connecting cable and a computer display providing feedback. The devices are end-driven and can provide movement in three dimensions. They offer various modes of active, passive, weight loss assisted, and direction-restricted active–passive movement, with movement feedback for each mode through a real-time situational interactive game.

### Intervention

All patients of two groups received a 60 min routine rehabilitation treatment, including physical therapy (neuromuscular electrical stimulation, rolling, transferring, sitting, standing and walking) and occupational therapy (dressing, eating, grooming). Based on the routine treatment, the BRT group received 30 min of bilateral robot-assisted training, while the CT group received 30 min of task-related training, including reaching to move a cup, grasping and releasing blocks, picking up coins, and rolling a bucket. All treatments were given once a day, 6 days /week, for a total of 3 weeks.

**Fig. 1:**
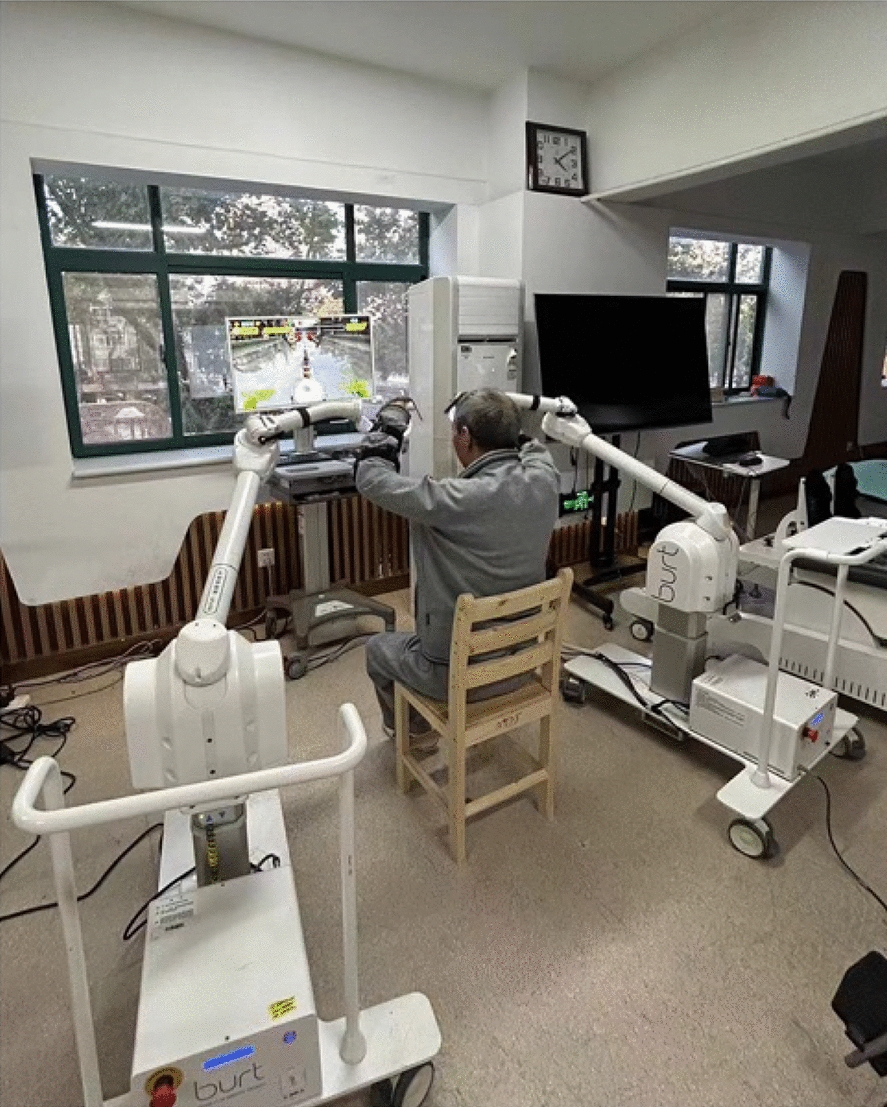
Bilateral upper limb robot-assisted training

For the bilateral upper limb robot-assisted therapy, the patient is seated in the treatment chair between two upper limb rehabilitation robots. The robots are then adjusted so that the blue line on the robot arm is aligned with the patient’s acromion. The upper limbs are secured to the end-effectors of the intelligent force feedback rehabilitation robot with glove straps to prevent slippage during treatment, and to secure the trunk. The training program was switched to bilateral arm training mode. The assistance force was adjusted to resist the gravity of arms. Then, the range of motion is calibrated according to the patient's active mobility or the therapist's prescription, and games with simultaneous bilateral movements are selected (drumming, flag waving, rowing), 30 min, respectively (Fig. 1).

### Outcome assessment

All outcome measures were assessed pre-(*T*1) and post-treatment (*T*2; at the end of the 3 week treatment). The primary outcome measure was the Fugl-Meyer assessment-Upper Extremities (FMA-UE). Secondary outcomes included the modified Barthel index (MBI) score, as well as quantitative electroencephalography (QEEG). The observation indexes of QEEG include power of electroencephalogram rhythms and brain functional connectivity.

### FMA-UE

FMA-UE was used to evaluate upper limb motor function. There were 33 assessment sub-items with a total score of 66 points. The score standard as follows: 0 indicates completely inactive; 1 indicates only part of the activities can be completed; 2 indicates the ability to perform activities normally. The higher the score, the better motor function.

### MBI

The MBI score was used to evaluate the ability to perform activities of daily living (ADL) including 10 functions, controlling urination, modification, toilet use, eating, bed and chair transfer, bathing, walking activities, dressing, and going up and down stairs. Full score = 100 points. A higher score reflects better independence, with a lower score reflecting stronger dependence.

### QEEG

**Fig. 2:**
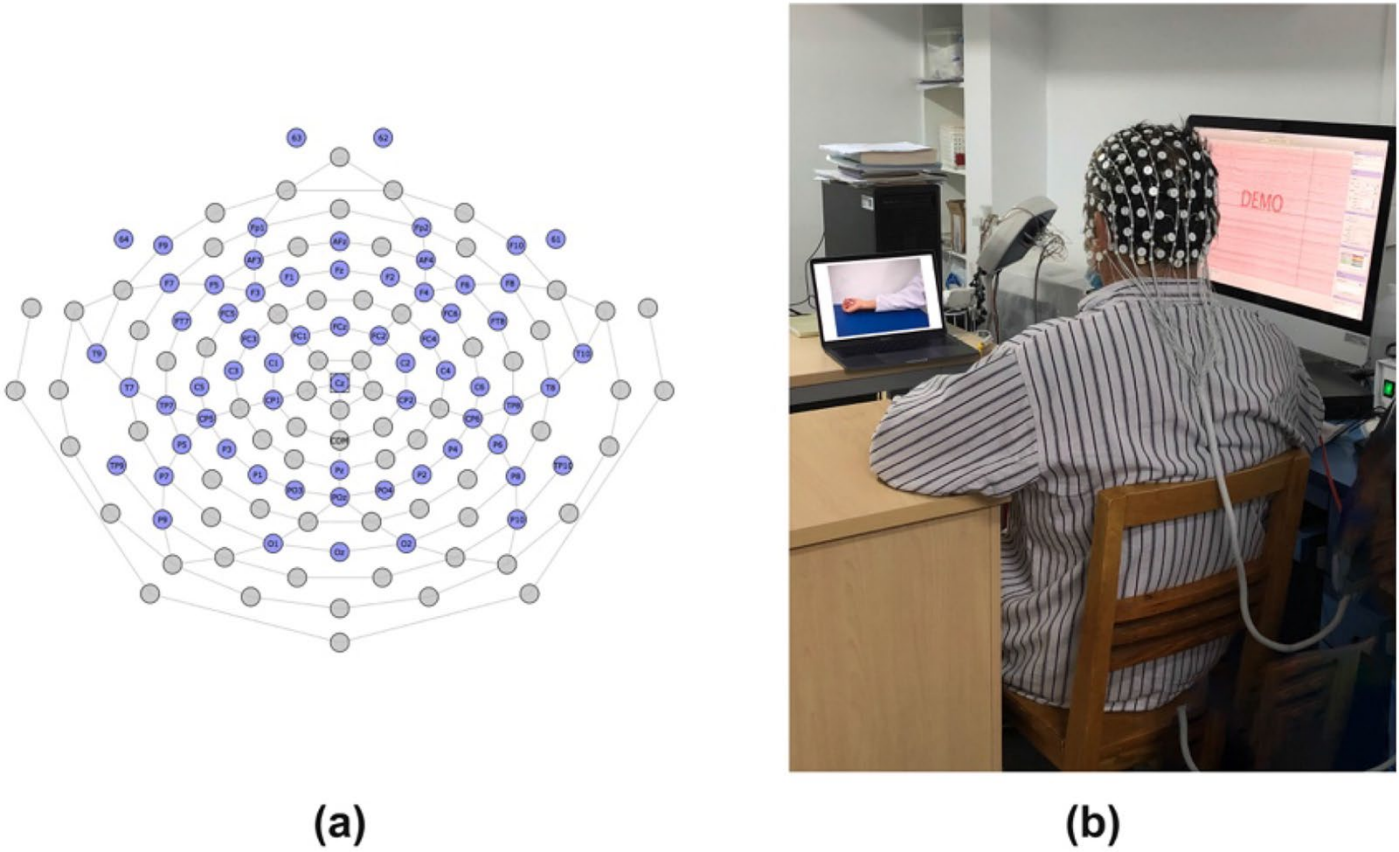
**a** The distribution map of 64-channel EEG electrodes. **b** EEG assessment

The 64-lead EEG system of the EGI company in the United States was used for EEG acquisition. A quiet, semi-dark, and electrically shielded examination room was selected. Subjects were asked to keep awake, quiet, closed eyed, and comfortably sitting after breakfast in the morning with tests completed by the same EEG examiner. In the recording of EEG, the Cz electrode in the center of the head was used as the reference electrode, and the sampling frequency was 1000 Hz. The impedance of each channel was lower than 5 kΩ, and the 50 Hz power frequency interference was filtered for five minutes. The data processing and analysis software Matlab2013a of THE MATH WORKS company and EEGLAB toolkit were used to analyze EEG data offline. The reference electrode was converted from the recorded Cz electrode to the average of all electrodes, and the band-pass filter was 0.05–30 Hz; segmentation was carried out in 3 s. An independent component analysis (ICA) algorithm was used to correct artifacts such as electrocardiogram and EMG. Hamming window and fast Fourier transform (FFT) were used to perform spectrum analysis on non-overlapping windows of EEG records obtained during the resting state. In the frequency spectrum density spectrum, power amplitude is represented by the square of each Hertz microvolt, and the absolute power is analyzed by FFT transform to obtain the frequency characteristics of different frequency bands. EEG frequency band division is as follows: *δ* (1.0–3.9 Hz), *θ* (4.0–7.9 Hz), *α* (8.0–13.9 Hz), *β* (14.0–30.0 Hz). Functional connectivity (FC) uses the HERMES toolkit to calculate a weighted phase-lag index (wPLI) of *α* and *β*. The calculation method of wPLI is as follows: Frequency bands of interest (alpha, beta) are defined. The EEG data of electrode *X* and electrode *Y* were filtered according to the above interested frequency bands. The filtered signals of electrode *X* and *Y* were transformed with a Hilbert transform, and the phase φx and φy at each time point were extracted. Finally, the cross spectrum was calculated, and the wPLI value of each segment was calculated for the average between segments. Among them, the wPLI value range was 0–1, the larger the value, the stronger the functional connection (Fig. 2).

### Statistical analysis

SPSS23.0 statistical software was used for statistical analysis. The measurement data with normal distribution and homogeneity of variance were expressed as (‾*x* ± *s*). The paired sample *t*-test was used for intra-group comparison, while the independent sample *t*-test was used for inter-group comparison. For count data that do not conform to normal distribution and homogeneity of variance, a Chi-square test was used, and the significance level was *p* < 0.05.

## Results

### Case data results

There were no significant differences in age, gender, course of disease, stroke type, or affected side between the two groups (Table 1).

**Table 1:** Baseline characteristics of the study subjects

	Bilateral group (*n* = 12)	Conventional group (*n* = 12)	*t*/*χ*2	*p* value
Age, year, mean (SD)	63.25(7.94)	60.58(6.33)	0.482	0.373
Sex, *n* (% male)	10 (83.33)	11 (91.67)	0.381	0.537
Time since stroke onset (weeks), mean (SD)	2.75(1.76)	3.33(1.78)	0.177	0.428
Type of stroke				
Ischemic	11	10	0.381	0.537
Hemorrhagic	1	2		
Site of stroke				
Subcortex	9	10	0.000	1.000
Mutiple	3	2		
Lesion site, *n* (%left)	6 (50)	5 (41.67)	0.168	0.682

### Motor performance and ADL

**Fig. 3:**
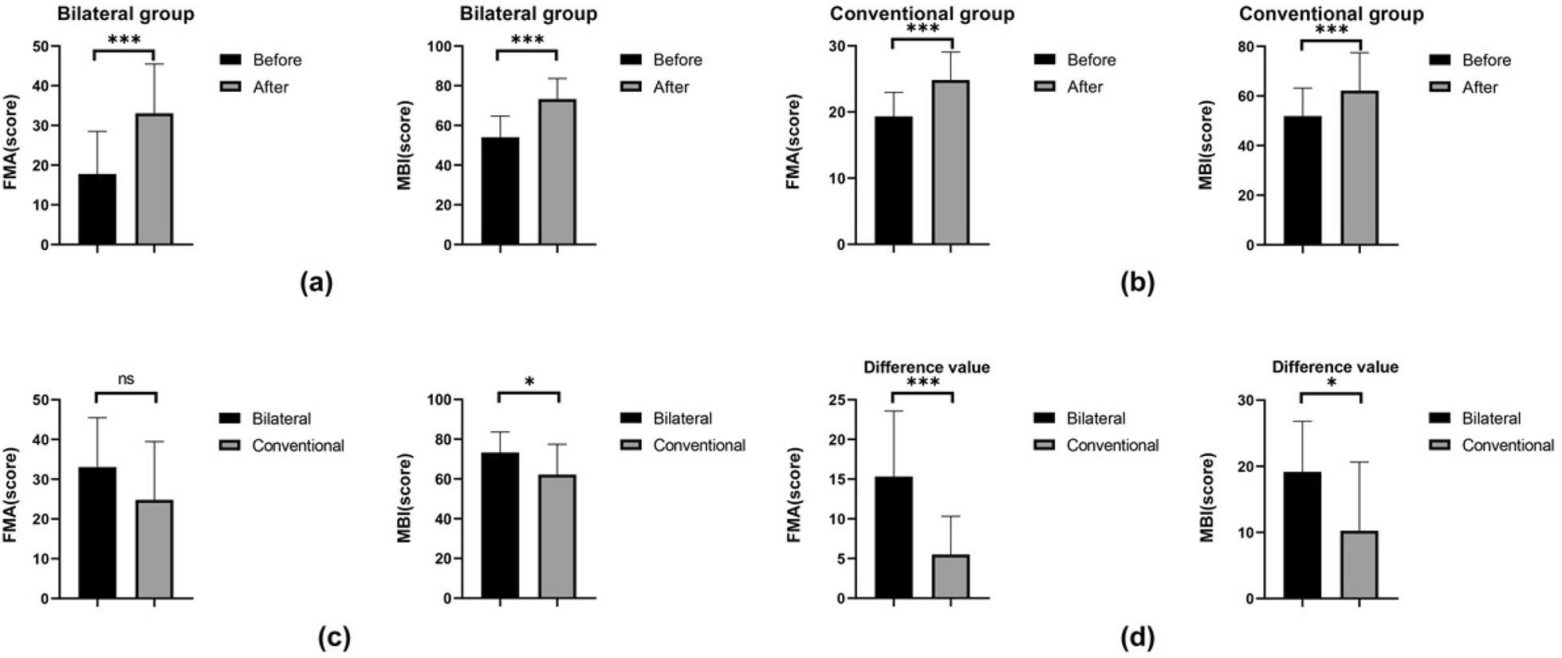
Motor performance and ADL in two groups. **a** FMA-UE and MBI scores before and after treatment in **BRT** group. **b** FMA-UE and MBI scores before and after treatment in **CT** group. **c** Comparison of FMA-UE and MBI scores after treatment between two groups. **d** Difference of FMA-UE and MBI scores before and after treatment between two groups. FMA-UE: Fugl-Meyer Assessment-Upper Extremities, MBI: Modified Barthel Index

As presented in Fig. 3, no significant differences in FMA-UE and MBI scores among the two groups before treatment existed (*p* > 0.05). Within-group differences in FMA-UE and MBI scores for both the bilateral and conventional groups were statistically significant after treatment. A between-group comparison indicated that the MBI score of the bilateral group was significantly higher than that of the conventional group, while the FMA-UE score was not significantly different from that of the conventional group. The differences of FMA-UE and MBI scores before and after treatment in the bilateral group was significantly different compare to those in the conventional group.

### Power of electroencephalogram rhythms

**Fig. 4:**
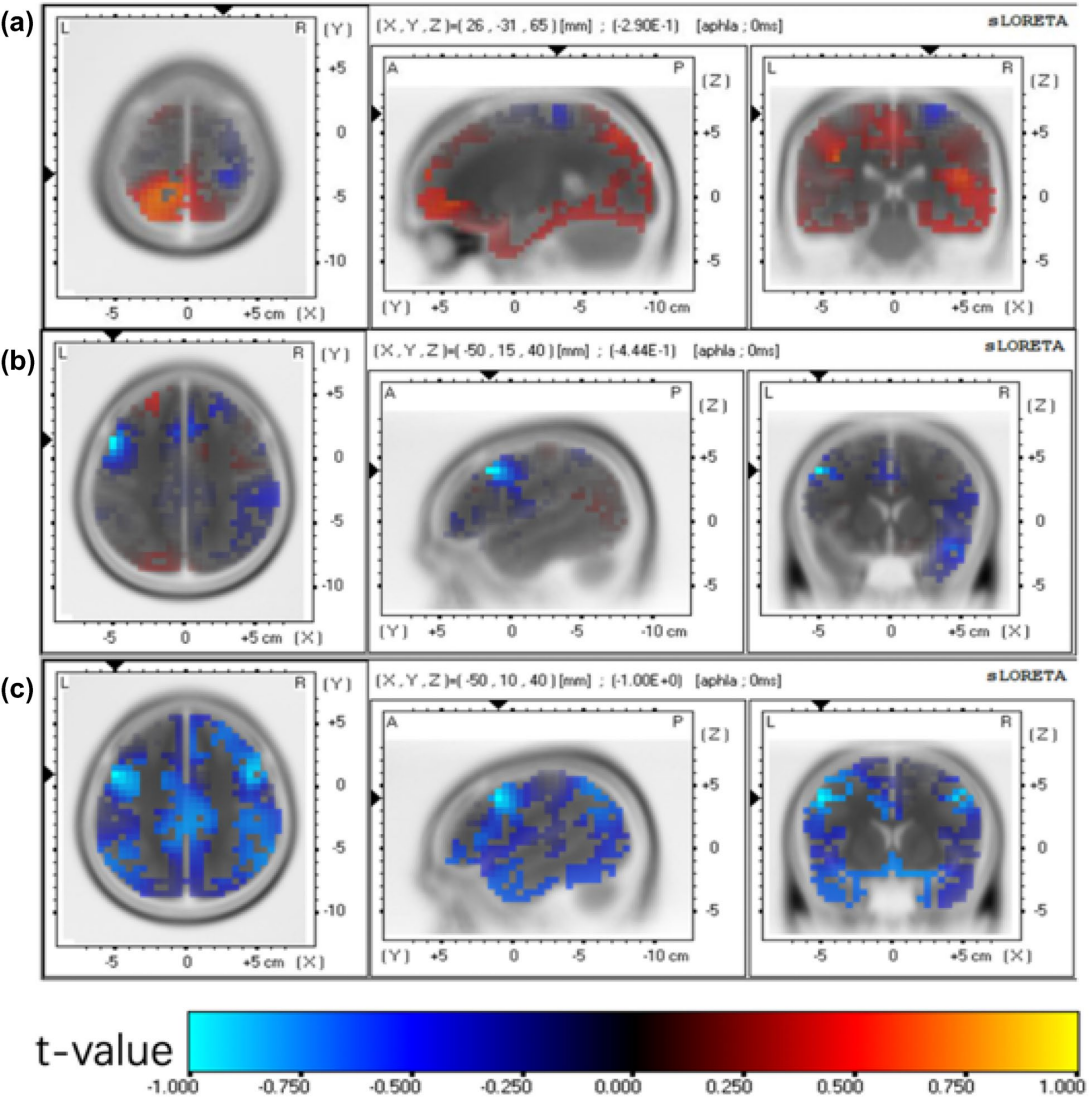
Current source density in two groups after *α* frequency treatment, **A** Current source density in **CT** group after treatment, **B** Current source density in **BRT** group after treatment, **C** Comparison of current source density between the two groups

**Fig. 5:**
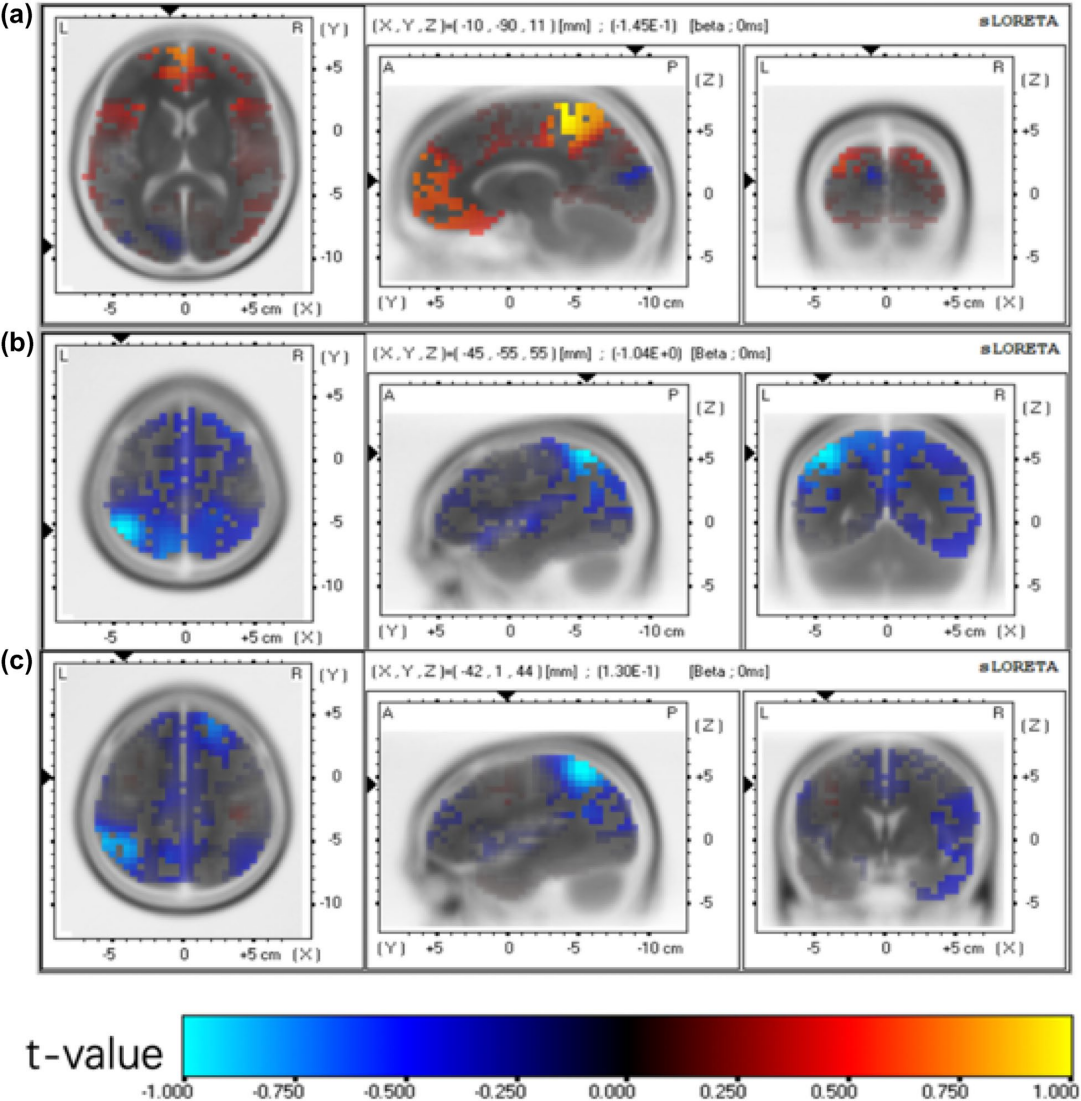
Current source density in two groups after *β* frequency treatment, **A** Current source density in CT group after treatment, **B** Current source density in BRT group after treatment, **C** Comparison of current source density between the two groups

The differences in the density of current sources at different frequencies between the two groups after treatment were observed, and the topographic maps of the statistical results are shown in Figs. **4**, **5**. From the alpha frequency map (Fig. 4), it can be seen that localized brain activity in the CT group after treatment was in the frontal lobes (BA4, BA6, BA9) (blue part in Fig. 4A); the sites of enhanced activity in the bilateral group after treatment were located in the paracentral lobule (BA2, BA3) and precentral gyrus (BA4, BA6) (blue part in Fig. 4B). The sites of enhanced activity in the BRT group compared with the CT group were located in the precentral gyrus (BA4, BA6) (blue part in Fig. 4C). As seen from the β-frequency diagram (Fig. 5), the localized brain activity in the CT group was in the parietal lobe (BA39) after treatment (blue part in Fig. 5A), while the enhanced activity in the BRT group was in the frontal lobe (BA4, BA6) and parietal lobe (BA7, BA40) bilaterally (blue part in Fig. 5B). The enhanced activity in the bilateral group was in the frontal lobe (BA4) and parietal lobe (BA5) bilaterally compared with the CT group (blue part in Fig. 5C).

### Brain functional connectivity

**Fig. 6:**
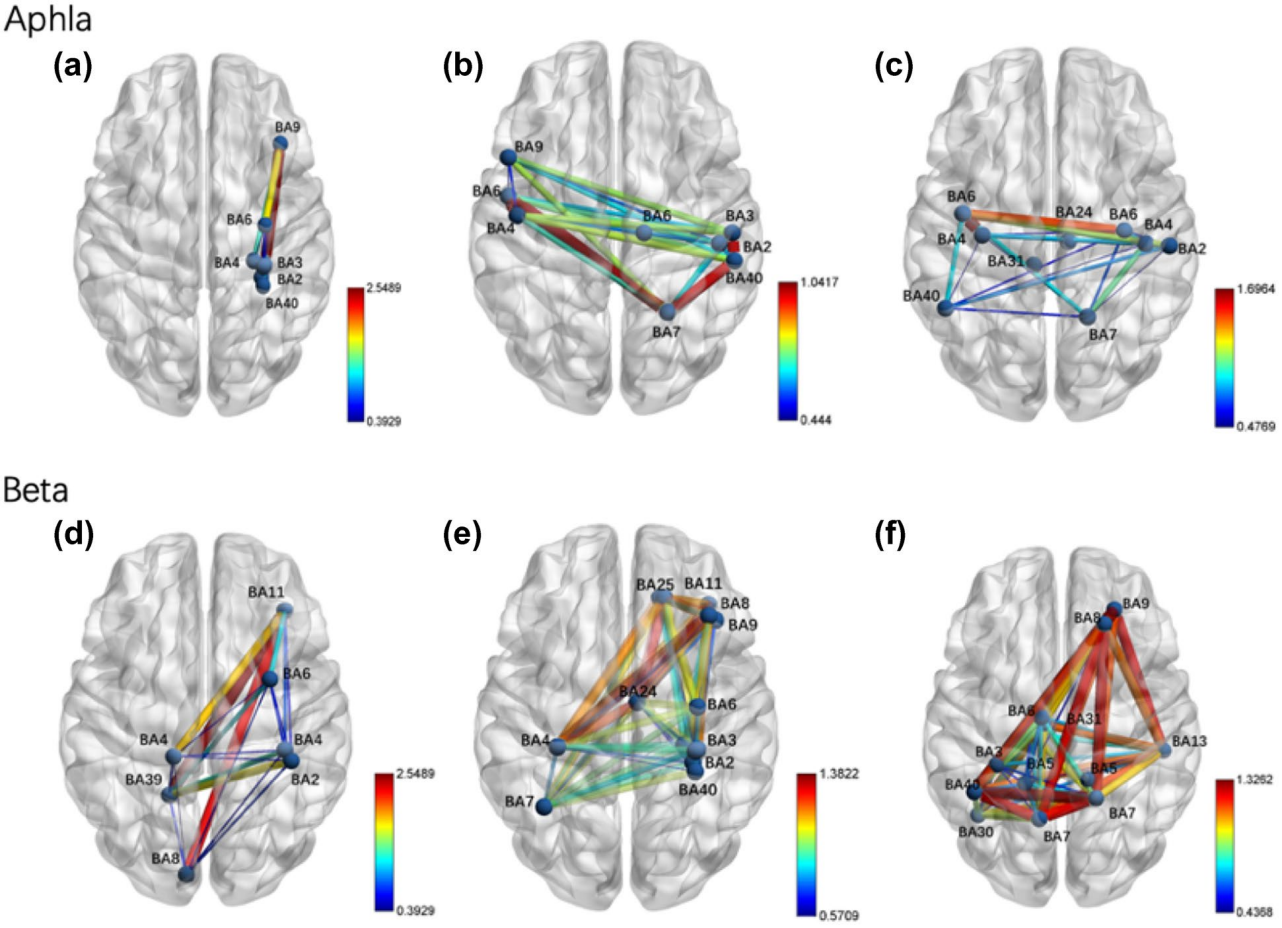
Topography of functional connectivity differences between the two groups after treatment. Alpha frequency: **A** After treatment in the CT group, **B** After BRT, **C** Comparison between the two groups; beta frequency: **D** After treatment in the **CT** group, **E** After **BRT**, **F** Comparison between the two groups

By comparing the brain functional connectivity constructed for the cortical regions of interest, it was found that at α frequency, the regions of interest (ROI) were BA4, BA6, BA7, BA40, BA31, and BA24. The brain functional connectivity was significantly different at the local frontal, limbic, and frontal–parietal connections (Fig. 6C), and this significant functional connectivity was more pronounced in the BRT group compared to the CT group. At β frequency, the regions of interest (ROI) were BA3, BA5, BA6, BA7, BA8, BA9, BA13, BA30, BA31, and BA40. There were significant differences in local parietal, frontal, and brain functional connectivity at local frontal, limbic, and frontal–limbic connections (Fig. 6F). This significant functional connectivity was also reflected in the cortical response after treatment in the BRT group.

## Discussion

Our study compared the effects of CT and BRT on upper limb function and activities of daily living in stroke patients. The results show BRT better improves patients' upper limb motor function and ADL performance than CT. In addition, this study examined the EEG cortical activity and network of the two groups of patients in an attempt to reveal potential neural mechanism differences between the different intervention methods. The results showed that there was a significant difference in the functional brain connectivity between the two groups of patients, both in terms of brain activity regions, as well as regions of interest (ROI), with the bilateral group showing better results than the CT group.

FMA-UE was the main outcome indicator in this study, and the results of the study showing no significant difference between the BRT and CT groups after treatment. However, the BRT group showed significantly greater improvement in FMA-UE difference before and after treatment compared to the CT group. Wu et al.'s meta-analysis showed no significant difference in the BRT group compared to the CT for the improvement of upper limb function in patients [23]. According to previous reports, BRT is more effective in patients with low function and severe motor impairment [24], and the relatively severe motor dysfunction of the patients included in the present study is consistent with those results. There was no statistical difference in the comparison of FMA between the two groups after treatment in this study, while there was a statistical difference in the comparison of the difference between the two groups before and after treatment. The reason for this may be unknown confounding factors in the clinical study and complete randomization of the groups could not be achieved. The difference was statistically significant, indicating to some extent that the BRT group had better treatment effects than the CT group.

A meta-analysis study in Cochrane also showed that upper limb robotics improved the ability to perform activities of daily living in patients with acute and subacute stroke, but not in patients with chronic stroke [25]. Lee et al. compared bilateral arm training with routine occupational therapy in 30 stroke patients for eight weeks and reported that the improvement in the ADL performance was greater in the group that received bilateral upper limb training than in the group that received routine occupational therapy [26], indicating bilateral training can be an effective means of functional training of the upper limbs in patients with chronic-phase stroke. However, a systematic review by Kwakkel et al. [27] showed no significant improvement in daily living abilities of stroke patients before and after treatment in the upper limb robotic training group, probably due to the large heterogeneity in the course of the disease of patients included in the meta-analysis, resulting in inconsistent results with subsequent studies.

Regarding the comparison of quantitative EEG, we found the power spectrum energy of the alpha rhythm was not reduced in the BRT group compared to the conventional group. By retrospective analysis, the functional brain connections in the region of interest (ROI) were significantly different between the two groups at the frontal, limbic, and parietal connections on both sides. Previous studies have also shown that changes in the coupling strength between hemispheres in homologous regions are associated with the degree of recovery of motor function, and this correlation mainly exists between primary motor regions [28–30], which may be one of the potential neural mechanisms that the BRT group has better efficacy than the CT group. Beta rhythms are usually associated with logical, complex thinking, and attention, which are closely related [31]. The results showed a significant increase in the power spectrum energy of the beta rhythm in the bilateral group after treatment, compared to the conventional group. Additionally, the functional brain connections in the region of interest (ROI) were significantly different in the bilateral frontal and limbic connections. This suggests that BRT not only mobilized more cognitive functions in the brain, but also significantly increased the connections between the primary motor area on the affected side and the supplementary motor area (SMA) on the contralateral side. Functional magnetic resonance studies have confirmed the early involvement of SMA in stroke recovery, and its initial activation is related to motor recovery [32, 33]. The Park study also found that the FC between primary motor cortex (M1) on the affected side, the contralateral thalamus, and SMA was positively correlated with motor function at 6 months [34]. A possible mechanism is that, in the case of greater serious motor impairment, secondary motor areas such as the premotor cortex (pMC) and SMA participate in the reorganization of motor networks to promote motor performance recovery [35, 36].

In the normal resting state, the brain shows an active network of interactions between the two hemispheres, and changes in resting-state activity in the cortex after stroke are associated with motor dysfunction during movement [37]. Moreover, in the present study, the electroencephalogram (EEG) results showed a significant increase in B4/B6 functional connectivity between the bilateral hemispheres after bilateral robotic therapy, suggesting the plasticity changes of the integrated brain functional network related to the recovery of upper limb movement after stroke [38]. Resting α activity reflecting levels of awareness and attention, memory, motor learning, and performance [39, 40], and is correlated with subacute clinical state, motor performance and functional recovery after stroke [41]. There is a positive correlation between whole-brain connectivity and motor recovery outcomes at 3 months in the beta frequency range at two to three weeks after stroke [42]. The extent of connectivity reorganization is related to the extent of motor recovery [43], the results showed that the related connectivity involved in recovery (or strengthening) has a positive impact on the subsequent recovery of upper limb motor, which provides us with a new opportunity to explore the functional connectivity of brain network after stroke in our future study.

This trial has several limitations. First, the study included patients with a disease duration of less than three months, and observed the effect of BRT on upper limb function. Further studies are needed to investigate the effect of BRT on stroke patients with a disease duration of three months or longer. Second, because of the epidemic, this study included a small sample size and did not observe the effects of unilateral upper extremity robotic therapy, and a larger sample is still needed for further studies in the future. Third, the observation period of this study was short and quantitative EEG evaluation before treatment was missing.

## Conclusion

Our findings suggest that the use of BRT in addition to conventional treatment improves ADL performance in stroke patients, but is not superior for improving motor function in patients. Quantitative EEG tests showed that BRT increased the connectivity of bilateral cerebral hemispheres, especially M1 and the contralateral SMA.

## Data Availability

The datasets generated and analyzed during the current study are available upon request.
